# Supplementary material for: Revealing 3D microanatomical structures of unlabeled thick cancer tissues using holotomography and virtual H&E staining
Source: Nat Commun. 2025 May 22;16:4781. doi: 10.1038/s41467-025-59820-0 (PMC12098747; doi:10.1038/s41467-025-59820-0)
Supplement: Supplementary file 1 — Supplementary Information [file 41467_2025_59820_MOESM1_ESM.pdf]

Supplementary information

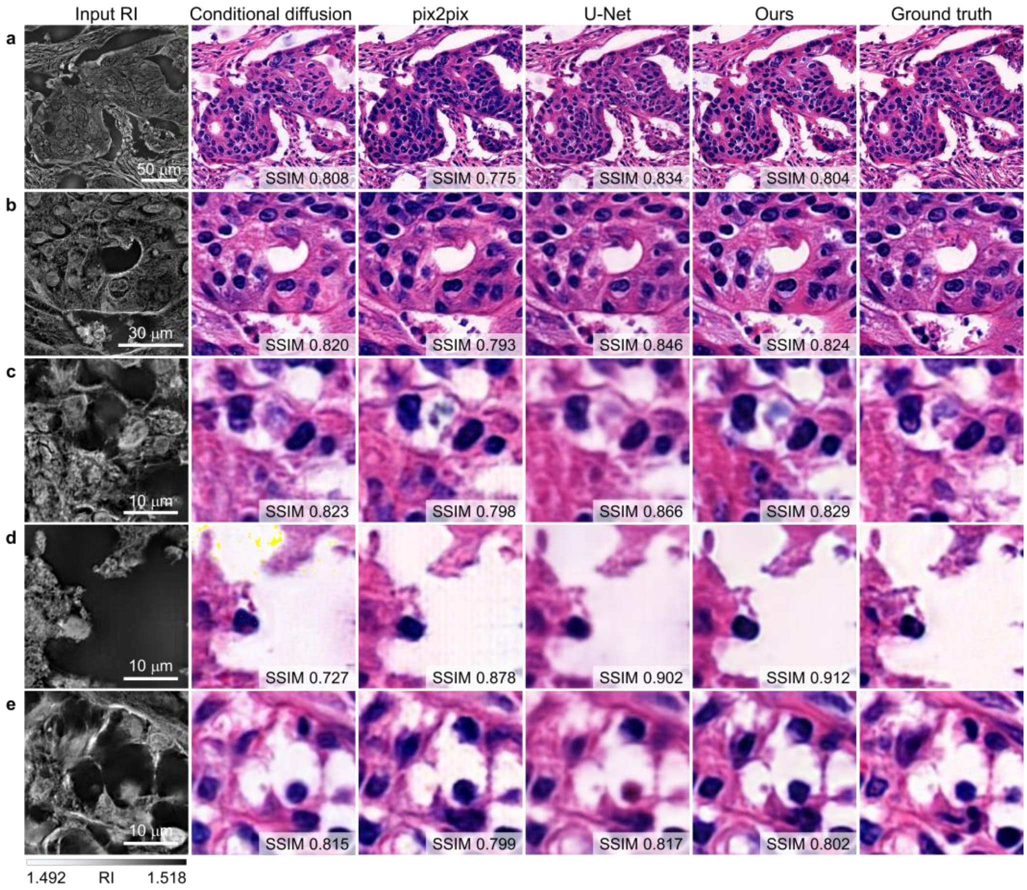

Supplementary Fig. 1| Comparing performance between various neural networks. Representative input RI images and corresponding virtual H&E images generated by conditional diffusion[1], pix2pix[2], U-Net[3], our model, and ground truth H&E images are shown in each column. SSIM values between virtual and ground truth images appear below each virtual H&E image. Results are from a single experiment.

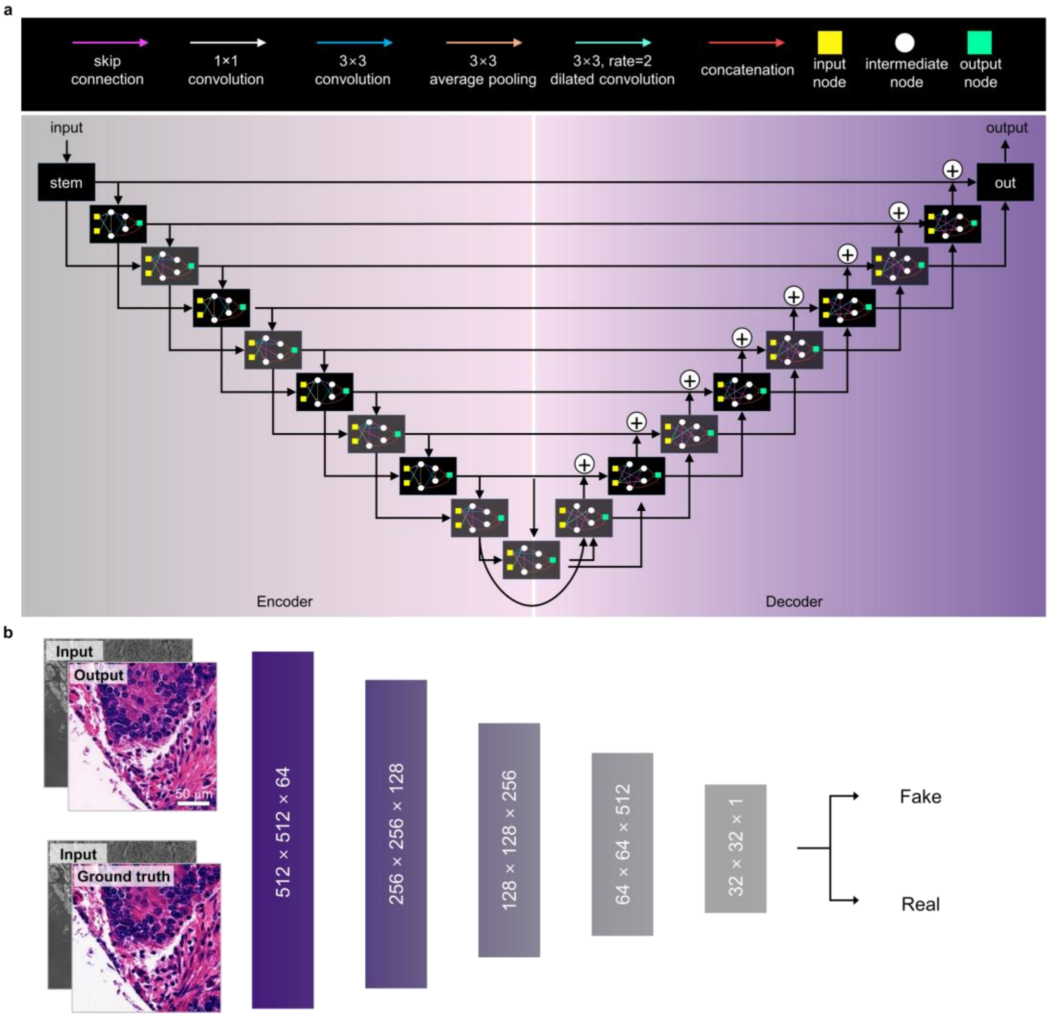

Supplementary Fig. 2| Network architecture. a, Architecture of the SCNAS used for generator[4]. The structure includes eight encoding micro-level architectures, a bridge, and another decoding eight micro-level architectures, resulting in a U-Net-like structure. **b,** Architecture of the discriminator. The structure is benchmarked from the previous work[2], which includes five sequential convolutional layers.

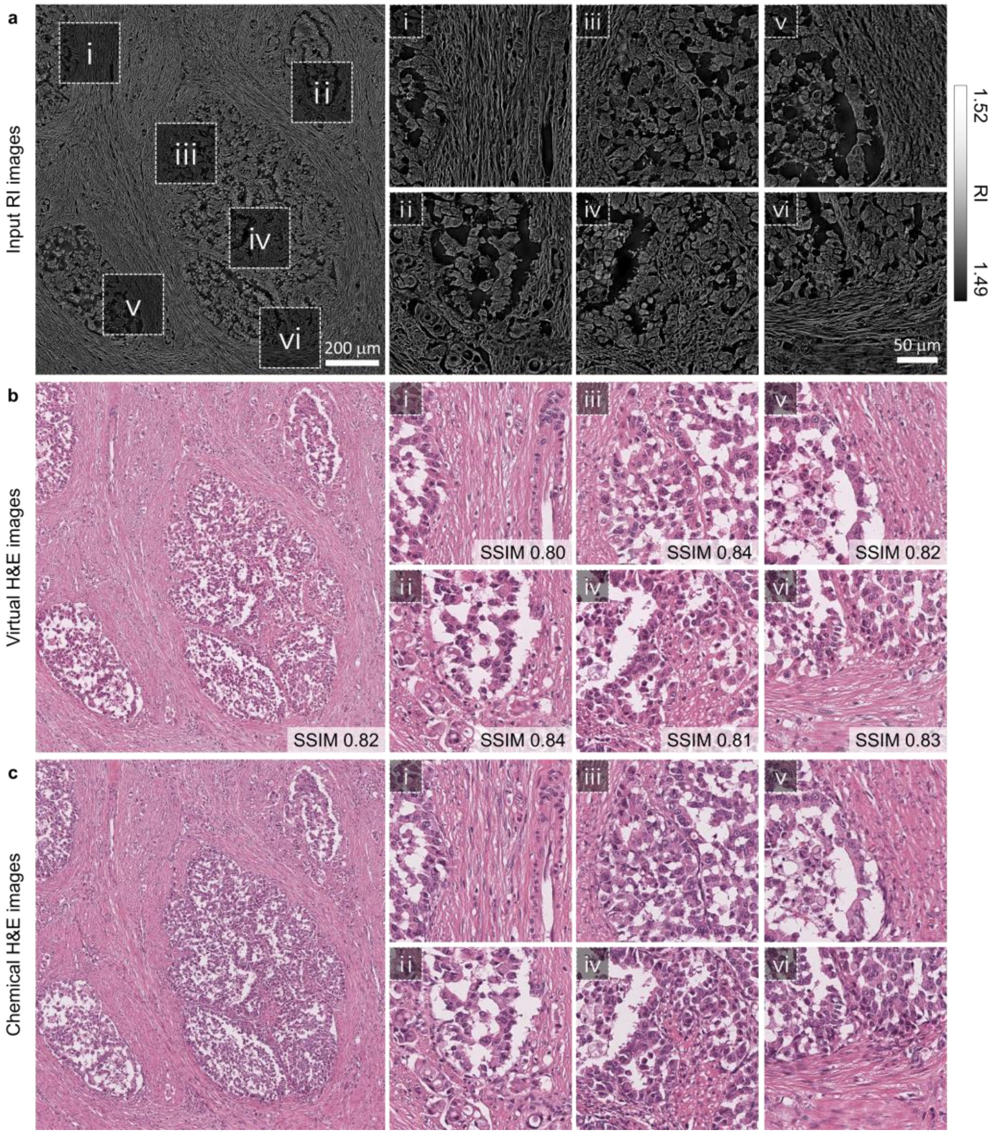

Supplementary Fig. 3| Validations of the trained network with a 5 μm-thick, H&E-stained gastric cancer tissue slide. a, A wide-field RI image obtained from a 5 μm-thick, H&E-stained gastric cancer slide and its detailed images. **b,** Wide-field H&E stained images generated by the trained neural network and its detailed images. **c,** Ground truth H&E images obtained using a WSS and its detailed images. Results are from a single experiment.

	U-Net	pix2pix	Conditional diffusion	Ours
SSIM	0.8056 ± 0.0236	0.7580 ± 0.0216	0.7754 ± 0.0248	0.7847 ± 0.0145
PSNR	18.2665 ± 1.7187	17.3047 ± 1.8176	11.3599 ± 1.3261	18.0068 ± 1.4969

Supplementary Table 1| Comparison of neural network performance metrics based on mean and standard deviation. Distribution of SSIM and PSNR values computed from 100 cropped patches.

References

1 Saharia, C. *et al.* Image Super-Resolution via Iterative Refinement. *IEEE Transactions on Pattern Analysis and Machine Intelligence* **45**, 4713-4726, doi:10.1109/TPAMI.2022.3204461 (2023).

2 Isola, P., Zhu, J.-Y., Zhou, T. & Efros, A. A. in *Proceedings of the IEEE conference on computer vision and pattern recognition.* 1125-1134.

3 Ronneberger, O., Fischer, P. & Brox, T. in *Medical image computing and computer-assisted intervention–MICCAI 2015: 18th international conference, Munich, Germany, October 5-9, 2015, proceedings, part III 18.* 234-241 (Springer).

4 Kim, S. *et al.* in *Medical Image Computing and Computer Assisted Intervention–MICCAI 2019: 22nd International Conference, Shenzhen, China, October 13–17, 2019, Proceedings, Part III 22.* 220-228 (Springer).
