# Supplementary material for: Revealing 3D microanatomical structures of unlabeled thick cancer tissues using holotomography and virtual H&E staining
Source: Nat Commun. 2025 May 22;16:4781. doi: 10.1038/s41467-025-59820-0 (PMC12098747; doi:10.1038/s41467-025-59820-0)
Supplement: Supplementary file 3 — Reporting Summary [file 41467_2025_59820_MOESM3_ESM.pdf]

nature portfolio

Corresponding author(s): YongKeun Park

Last updated by author(s): May 6, 2025

Reporting Summary

Nature Portfolio wishes to improve the reproducibility of the work that we publish. This form provides structure for consistency and transparency in reporting. For further information on Nature Portfolio policies, see our Editorial Policies and the Editorial Policy Checklist.

Statistics

For all statistical analyses, confirm that the following items are present in the figure legend, table legend, main text, or Methods section.

n/a	Confirmed	
☐	☒	The exact sample size (*n*) for each experimental group/condition, given as a discrete number and unit of measurement
☐	☒	A statement on whether measurements were taken from distinct samples or whether the same sample was measured repeatedly
☐	☒	The statistical test(s) used AND whether they are one- or two-sided<br>*Only common tests should be described solely by name; describe more complex techniques in the Methods section.*
☒	☐	A description of all covariates tested
☒	☐	A description of any assumptions or corrections, such as tests of normality and adjustment for multiple comparisons
☐	☒	A full description of the statistical parameters including central tendency (e.g. means) or other basic estimates (e.g. regression coefficient) AND variation (e.g. standard deviation) or associated estimates of uncertainty (e.g. confidence intervals)
☐	☒	For null hypothesis testing, the test statistic (e.g. *F*, *t*, *r*) with confidence intervals, effect sizes, degrees of freedom and *P* value noted<br>*Give P values as exact values whenever suitable.*
☒	☐	For Bayesian analysis, information on the choice of priors and Markov chain Monte Carlo settings
☒	☐	For hierarchical and complex designs, identification of the appropriate level for tests and full reporting of outcomes
☐	☐	Estimates of effect sizes (e.g. Cohen's *d*, Pearson's *r*), indicating how they were calculated

Our web collection on statistics for biologists contains articles on many of the points above.

Software and code

Policy information about availability of computer code

Data collection	A commercial software, HTX processing server version 1.5.20, was used to obtain the holotomography dataset.
Data analysis	HoVer-Net, an open-source software, was utilized for nuclei segmentation using a pre-trained model based on the CoNSeP dataset. For dataset pre-processing, a customized code, developed based on a previously published paper, was implemented using MATLAB (R2021b) and Python (3.8.0). The virtual staining, a key task in this study, was performed using another customized code trained in pytorch (1.13.1). The deep learning models involved were built using standard libraries and scripts available in PyTorch, with the code for our virtual staining framework accessible at this GitHub repository (https://github.com/BMOLKAIST/3D_virtual_HE_staining). Additionally, the open-source software ImageJ, specifically the "Grid/Collection stitching" plugin, was used for image stitching. Statistical analyses were conducted using MATLAB (R2021b).

For manuscripts utilizing custom algorithms or software that are central to the research but not yet described in published literature, software must be made available to editors and reviewers. We strongly encourage code deposition in a community repository (e.g. GitHub). See the Nature Portfolio guidelines for submitting code & software for further information.

Data

Policy information about availability of data

All manuscripts must include a data availability statement. This statement should provide the following information, where applicable:

- Accession codes, unique identifiers, or web links for publicly available datasets
- A description of any restrictions on data availability
- For clinical datasets or third party data, please ensure that the statement adheres to our policy

The training, testing, and exemplary data used in this study are available in the science database https://doi.org/10.57760/sciencedb.24217

Research involving human participants, their data, or biological material

Policy information about studies with human participants or human data. See also policy information about sex, gender (identity/presentation), and sexual orientation and race, ethnicity and racism.

Reporting on sex and gender	The histology slides used in this study were obtained under IRB protocols that protect patient privacy, in accordance with the ethical standards of the Gangnam Severance Hospital (IRB No. 3-2022-0083) and Seoul National University Boramae Medical Center (IRB No. 30-2018-32). As a result, information such as sex, gender, race, ethnicity, and other personal details cannot be provided.
Reporting on race, ethnicity, or other socially relevant groupings	The histology slides used in this study were obtained under IRB protocols that protect patient privacy, in accordance with the ethical standards of the Gangnam Severance Hospital (IRB No. 3-2022-0083) and Seoul National University Boramae Medical Center (IRB No. 30-2018-32). As a result, information such as sex, gender, race, ethnicity, and other personal details cannot be provided.
Population characteristics	N/A
Recruitment	N/A
Ethics oversight	This study complied with all relevant ethical regulations and was approved by the Institutional Review Board (IRB) of Gangnam Severance Hospital (IRB No. 3-2022-0083) and Seoul National University Boramae Medical Center (IRB No. 30-2018-32). Written informed consent was waived by the IRB due to the retrospective nature of the study and the use of residual samples without any clinical data collection.

Note that full information on the approval of the study protocol must also be provided in the manuscript.

Field-specific reporting

Please select the one below that is the best fit for your research. If you are not sure, read the appropriate sections before making your selection.

☒ Life sciences ☐ Behavioural & social sciences ☐ Ecological, evolutionary & environmental sciences

For a reference copy of the document with all sections, see nature.com/documents/nr-reporting-summary-flat.pdf

Life sciences study design

All studies must disclose on these points even when the disclosure is negative.

Sample size	A colon cancer slide was obtained to train the colon virtual staining neural network. Four gastric cancer slides from different patients were used to train the gastric virtual staining neural network. For testing, label-free thick tissue slides (10, 20, and 50 µm) were obtained from different colon cancer patients, and a 20 µm label-free gastric tissue slide was obtained from a single gastric cancer patient.<br><br>Regarding the neural network training, e prepared a training dataset with more than 1,000 patches to ensure sufficient data for effective network training. Since the neural network was successfully trained and demonstrated robust performance on test datasets, we believe the dataset size exceeded the minimum required for optimal model performance.
Data exclusions	We did not exclude any data during the analysis.
Replication	We replicated the entire workflow—from imaging and training to testing—described in the paper twice: once for colon cancer slides and once for gastric cancer slides.
Randomization	All slides were reviewed and randomly selected by pathologists.
Blinding	All evaluations of the virtual staining results generated by the deep neural network were conducted blindly on tissue images that were not part of the training or validation phases.

Reporting for specific materials, systems and methods

We require information from authors about some types of materials, experimental systems and methods used in many studies. Here, indicate whether each material, system or method listed is relevant to your study. If you are not sure if a list item applies to your research, read the appropriate section before selecting a response.

Materials & experimental systems

n/a	Involved in the study
☒	☐ Antibodies
☒	☐ Eukaryotic cell lines
☒	☐ Palaeontology and archaeology
☒	☐ Animals and other organisms
☒	☐ Clinical data
☒	☐ Dual use research of concern
☒	☐ Plants

Methods

n/a	Involved in the study
☒	☐ ChIP-seq
☒	☐ Flow cytometry
☒	☐ MRI-based neuroimaging

Plants

Seed stocks	*Report on the source of all seed stocks or other plant material used. If applicable, state the seed stock centre and catalogue number. If plant specimens were collected from the field, describe the collection location, date and sampling procedures.*
Novel plant genotypes	*Describe the methods by which all novel plant genotypes were produced. This includes those generated by transgenic approaches, gene editing, chemical/radiation-based mutagenesis and hybridization. For transgenic lines, describe the transformation method, the number of independent lines analyzed and the generation upon which experiments were performed. For gene-edited lines, describe the editor used, the endogenous sequence targeted for editing, the targeting guide RNA sequence (if applicable) and how the editor was applied.*
Authentication	*Describe any authentication procedures for each seed stock used or novel genotype generated. Describe any experiments used to assess the effect of a mutation and, where applicable, how potential secondary effects (e.g. second site T-DNA insertions, mosiacism, off-target gene editing) were examined.*
